# Golden 2-like Transcription Factors Regulate Photosynthesis under UV-B Stress by Regulating the Calvin Cycle

**DOI:** 10.3390/plants13131856

**Published:** 2024-07-05

**Authors:** Xiangru Zhou, Wang Yu, Fushuai Gong, Hongwei Xu, Jie Lyu, Xiaofu Zhou

**Affiliations:** 1 Jilin Provincial Key Laboratory of Plant Resource Science and Green Production, Jilin Normal University, Siping 136000, China; 2 Faculty of Biological Science and Technology, Baotou Teachers’ College, Baotou 014030, China

**Keywords:** *Rhododendron chrysanthum* Pall., G2-like transcription factor, photosynthesis, chlorophyll fluorescence, multi-omics, UV-B stress

## Abstract

UV-B stress can affect plant growth at different levels, and although there is a multitude of evidence confirming the effects of UV-B radiation on plant photosynthesis, there are fewer studies using physiological assays in combination with multi-omics to investigate photosynthesis in alpine plants under stressful environments. Golden 2-like (G2-like/GLK) transcription factors (TFs) are highly conserved during evolution and may be associated with abiotic stress. In this paper, we used Handy-PEA and Imaging-PAM Maxi to detect chlorophyll fluorescence in leaves of *Rhododendron chrysanthum* Pall. (*R. chrysanthum*) after UV-B stress, and we also investigated the effect of abscisic acid (ABA) on photosynthesis in plants under stress environments. We used a combination of proteomics, widely targeted metabolomics, and transcriptomics to study the changes of photosynthesis-related substances after UV-B stress. The results showed that UV-B stress was able to impair the donor side of photosystem II (PSII), inhibit electron transfer and weaken photosynthesis, and abscisic acid was able to alleviate the damage caused by UV-B stress to the photosynthetic apparatus. Significant changes in G2-like transcription factors occurred in *R. chrysanthum* after UV-B stress, and differentially expressed genes localized in the Calvin cycle were strongly correlated with members of the G2-like TF family. Multi-omics assays and physiological measurements together revealed that G2-like TFs can influence photosynthesis in *R. chrysanthum* under UV-B stress by regulating the Calvin cycle. This paper provides insights into the study of photosynthesis in plants under stress, and is conducive to the adoption of measures to improve photosynthesis in plants under stress to increase yield.

## 1. Introduction

The reduction of the ozone layer has increased the amount of UV-B radiation (280–315 nm) reaching the Earth’s surface, posing a major threat to agricultural production [1]. Despite measures to reduce the use of ozone-depleting substances, UV-B radiation levels will increase in the future [2]. UV-B radiation is capable of affecting the normal growth of terrestrial plants, causing molecular damage and impacting on plant physiology and morphology, and is capable of reducing crop yields [1]. UV-B radiation can cause changes in plant morphology, such as leaf thickening, leaf curling, reduced stem elongation, and changes in root-crown ratio [3]. High-intensity UV-B radiation can cause DNA damage, produce reactive oxygen species, and impair photosynthesis [4].

The effects of UV-B radiation on the photosynthetic organs of green plants are manifested in causing damage to the oxygen release complex (OEC), D1/D2 reaction center proteins, and photosystem II (PSII), while reactive oxygen species are able to participate in UV-B-induced responses in plants [5]. It has been found that phosphorylation of serine 402 can alter UV RESISTANCE LOCUS 8 (UVR8) activity and promote flavonoid biosynthesis, facilitating adaptation to UV-B radiation in *Arabidopsis* [6]. The study of photosynthesis-related processes in plants under UV-B radiation may contribute to the understanding of photosynthesis-related mechanisms in plants in response to UV-B stress, which is important for reducing the problem of crop yield reduction under radiation conditions.

Some studies have found that *Rhododendron chrysanthum* Pall. (*R. chrysanthum*) growing in alpine environments are able to withstand harsh environments by increasing the activity and ratio of antioxidant enzymes [7]. *R. chrysanthum* growing on Changbai Mountain has been growing for a long time in an alpine tundra environment with high solar radiation, and its growth is exposed to UV-B stress. A study using metabolomics and proteomics to analyze the substance changes in leaves of *R. chrysanthum* under UV-B stress found that UV-B radiation can convert primary metabolites to phenolics and that abscisic acid may act synergistically with UV-B in the process of substance conversion [8]. Proteomics and acetylation proteomics studies showed that UV-B stress can cause acetylation modification of some photosynthetic proteins of *R. chrysanthum*, which in turn mitigates the damage caused by stress to the plant [9]. The adaptability of *R. chrysanthum* to intense UV radiation makes it a good experimental material for studying plant resistance to UV-B radiation.

Chloroplasts are the site of photosynthesis in plants and are able to dynamically adjust their energy conversion and metabolic properties according to the needs of plant metabolism and the environment [10]. The light reaction occurs in the membrane of the cystoid, where photosynthetic pigments are able to utilize the energy of sun photons to photolyze water to produce oxygen, and to provide ATP and reduced hydrogen for the Calvin cycle. Golden 2-like (GLK) transcription factors (TFs) are members of the GARP (Golden 2, ARR, and Psr1) superfamily and can play a central role in regulating chloroplast development. Most G2-like genes have two structural domains, including an Myb-DNA-binding structural domain (DBD, which contains an HLH motif) and a C-terminal structural domain (which contains a conserved GCT box) [11]. Studies on *Arabidopsis thaliana* have shown that GLK proteins are able to interact with promoter sequences to affect genes related to light trapping and chlorophyll biosynthesis, influencing photosynthesis in plants [12]. GLK proteins are able to regulate genes involved in light trapping and the vesicle-like protein complex, and may play a role in regulating the balance between the light and dark reactions of photosynthesis [13].

The development of “omics” technologies has allowed for a deeper understanding of the molecular mechanisms that regulate stress responses by identifying genes and proteins involved in regulating, adapting to, and influencing photosynthesis processes [14]. Analysis of reeds subjected to Cu stress using proteomics revealed that Cu stress reduced the content of chlorophyll a and chlorophyll b in reed leaves, while down-regulating the photosynthetic proteins PsbD, PsbO, and PsaA, which inhibited photosynthesis in reeds [15]. Metabolomics and transcriptomics studies indicate that galactinol regulates stomatal closure to control photosynthetic activity in sorghum during post-flowering drought [16]. Multi-omics analysis is able to integrate information from several histological levels to explain the same physiological process from different levels, and provide a deeper understanding of the molecular mechanisms of plant stress tolerance processes.

Chlorophyll fluorescence technology can determine the process of absorption, transmission, dissipation, and distribution of light energy in plant leaves, which can reflect the internal photosynthetic physiological characteristics of the crop, and realize the monitoring of photosynthetic physiology of the crop under the adversity, which is an effective method to study the plant resistance. In vivo rapid chlorophyll fluorescence rise kinetics enables the assessment of plant photosynthesis by non-invasive methods and has been widely used to monitor changes in the photosynthetic apparatus under different stresses [17,18]. Chlorophyll fluorescence imaging reflects the performance of the photosynthetic electron transport chain in plants and is a powerful tool for studying plant stress in vivo [19]. Therefore, in this study, changes in photosynthesis of *R. chrysanthum* after UV-B stress were determined using Imaging-PAM chlorophyll fluorescence imaging system (Imaging-PAM Maxi) and the plant efficiency analyzer (Handy PEA).

In the present study, *R. chrysanthum* group seedlings were used as experimental material for radiation treatment. The changes of photosynthetic parameters in *R. chrysanthum* after UV-B stress and abscisic acid treatment were investigated by using a chlorophyll fluorescence imaging system and the plant efficiency analyzer, and the changes of its photosynthetic physiology after UV-B stress were investigated using multi-omics. This study can enrich the understanding of the effect of UV-B stress on plant photosynthesis, which is conducive to enriching the theory of plant stress tolerance.

## 2. Results

As per the experimental design, *R. chrysanthum* was divided into four groups, Groups A and B were cultured in 1/4 MS medium and Groups C and D were cultured in 1/4 MS medium pre-applied with ABA. After 2 days (8 h/day) of continuous UV-B radiation (see Materials and Methods section for the radiation procedure), physiological and multi-omic tests were performed on the *R. chrysanthum* in different treatment groups.

### 2.1. UV-B Stress Impairs the Donor Side of Photosystem II in R. chrysanthum

**Figure 1:**
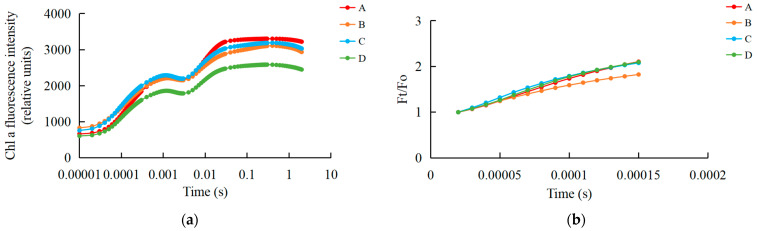

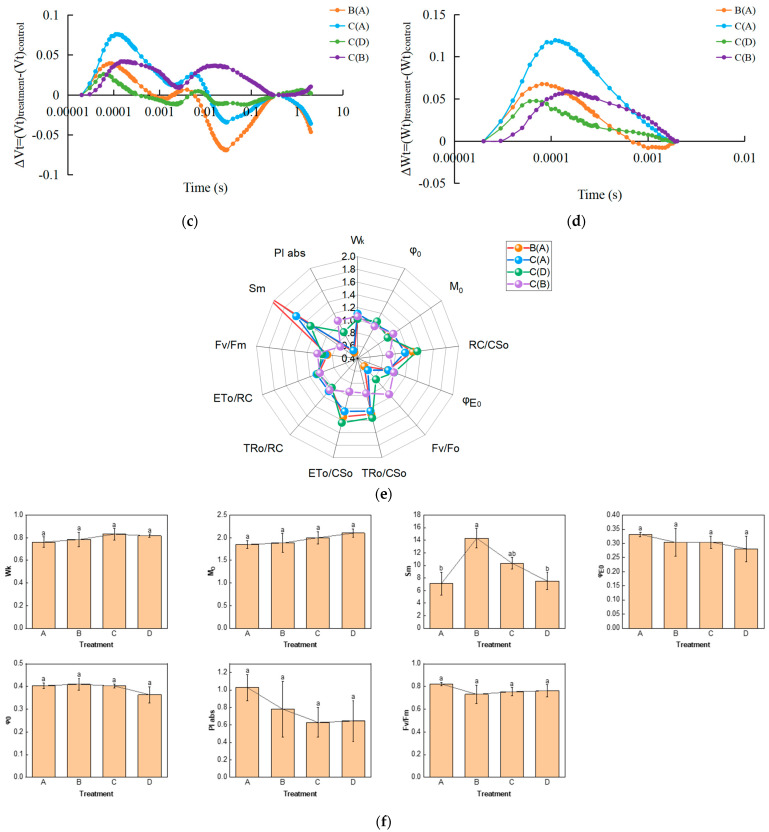
Kinetic characterization of OJIP transients in leaves of the *R. chrysanthum* following UV-B stress. (**a**) Fast chlorophyll fluorescence-induced kinetic curves; (**b**) O-point standardization; (**c**) Difference ΔVt curve between treatment and control Vt curves: orange and blue curves are for treatment A as control, green curves are for treatment D as control, purple curves are for treatment B as control; (**d**) Difference ΔWt curve between treatment and control Wt curves: orange and blue curves are for treatment A as control, green curves are for treatment D as control, purple curves are for treatment B as control; (**e**) Radar chart of fast chlorophyll fluorescence parameters; (**f**) Fluorescence parameters associated with PSII. Small letters a and b indicate significant differences (*p* < 0.05). Values are means ± S.E. (n = 3) based on analyses by one-way ANOVAs followed by LSD test. A: control; B: UV-B treatment; C: UV-B + ABA treatment; D: ABA treatment.

We examined the fast chlorophyll fluorescence-induced kinetic curves of *R. chrysanthum* leaves under different treatments using Handy-PEA, and the results are shown in Figure 1. The original O-J-I-P kinetic curve (OJIP maps) obtained from the assay are shown in Figure 1a, where the leaves exposed to UV-B radiation (group B) had their fluorescence reduced compared with control leaves (group A), while the leaves with pre-applied ABA (group C) had their fluorescence values in the middle of groups A and B. The results of normalization performed at point O (the minimum fluorescence value) are shown in Figure 1b. As only the original OJIP curve (Figure 1a) was analyzed, the amount of information obtained was small. The information of the OJIP curve is mainly embedded in the “shape” of the curve and the relative fluorescence intensity of the main characteristic points. In order to further interpret the OJIP curves, the curves were normalized to the O-P segment (Figure S1a) and to the O-J segment (Figure S1b). In the normalized O-P segment curves, the 2 ms and 30 ms positions correspond to fluorescence at the J and I points. In order to show the fluorescence values of the characteristic sites more clearly, the Vt curves of the treated and control samples were analyzed by difference to obtain the ΔVt curves (Figure 1c). The highest point of ΔVt indicates the primary site in the electron transport chain that suffers damage. The characteristic point between the O and J points, at about 300 μs, is called K. The elevation of the K point reflects the activity of the PSII donor side, especially the oxygen-excreting complex. To better compare the magnitude of damage suffered by the oxygen-excreting complex in treated and control leaves, ΔWt curves were produced (Figure 1d). With ΔWk > 0, UV-B stress resulted in a significant increase in the K point, indicating a significant inactivation of the PSII donor side of the oxygen-exploding complex.

The quantitative analysis based on the parameters obtained from the derivation of fast fluorescence curves can indirectly understand the PSII primary photochemical reactions, the electron transfer state, the activity and redox state of each reaction site of PSII, and assess the photosynthetic performance of *R. chrysanthum* leaves. We screened representative photosynthetic parameters (including those related to the receptor side and donor side of PSII) and plotted radar diagrams (Figure 1e; Table S1). In order to show more clearly the changes in the feature parameters after processing, we have used a histogram (Figure 1f; Table S1). The decrease in Mo after UV-B stress indicates that the rate of Q_A_ (primary quinone receptor) reduction during O-J was reduced, whereas preapplication of ABA was able to increase the rate of Q_A_ reduction. Sm was elevated after UV-B stress, indicating an increase in the plastoquinone (PQ) pool. And the results of BvsC group showed that ABA was able to reduce the PQ pool. The decrease in PIabs after UV-B stress indicated that the photosynthetic apparatus of leaves was damaged, whereas the results of BvsC group showed that ABA was able to reduce the damage of photosynthetic apparatus. By combining the four groups of treatments, it was found that there was little difference in photosynthesis between the treatment group applying ABA and the control group in the absence of UV-B radiation, whereas UV-B stress reduced photosynthesis by damaging the photosynthetic apparatus of the leaves, and the application of ABA was able to mitigate the damage to the photosynthetic apparatus caused by UV-B stress to a certain extent, with the most pronounced performance of the Sm parameter under the different treatments. Each photosynthetic parameter and its biological significance are shown in Table 1.

### 2.2. UV-B Stress Affects Photosynthesis in Leaves of R. chrysanthum

**Figure 2:**
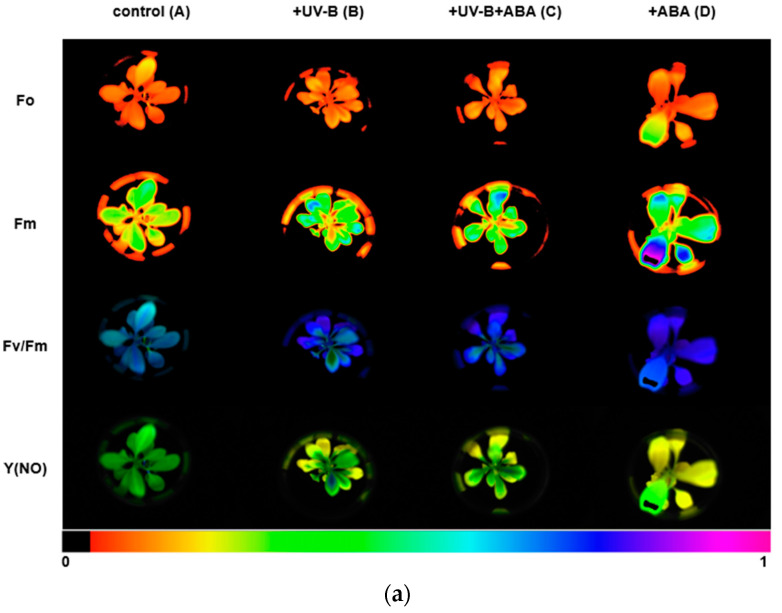

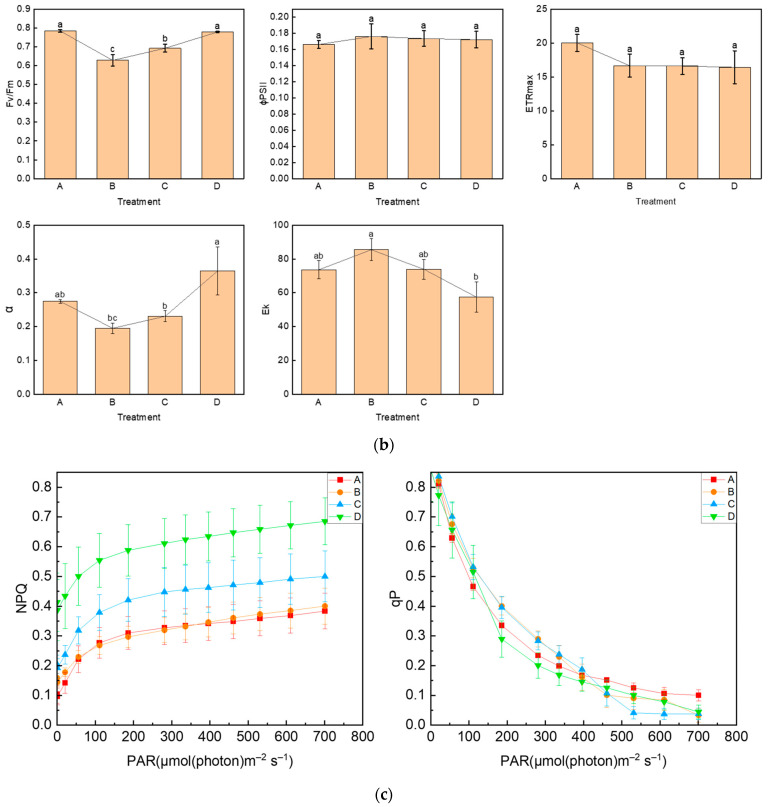
UV-B stress diminishes photosynthesis in *R. chrysanthum*. (**a**) Real-Time fluorescence imaging of *R. chrysanthum* leaves; (**b**) Fv/Fm, ΦPSII, ETRmax, α and Ek; (**c**) NPQ and qP as functions of PAR. Small letters a and b indicate significant differences (*p* < 0.05). Values are means ± S.E. (n = 3) based on analyses by one-way ANOVAs followed by LSD test. A: control; B: UV-B treatment; C: UV-B + ABA treatment; D: ABA treatment.

We used the Imaging-PAM chlorophyll fluorescence imaging system to measure chlorophyll fluorescence parameters. The images of Fo, Fm, Fv/Fm, and Y(NO) between different treatments are shown in Figure 2a, and the lower color bar from left to right indicates that the fluorescence value changes from small to large. In order to compare the changes in photosynthetic parameters between treatment groups, bar graphs of key parameters were presented (Figure 2b). After UV-B stress, Fv/Fm was significantly reduced and the maximum photosynthetic rate of photosystem II decreased. In contrast, Fv/Fm increased in group C, compared with group B, with preapplication of ABA, indicating that ABA could alleviate the effect of UV-B on the photosystem of *R. chrysanthum*. The initial slope after curve fitting is α, which can reflect the efficiency of photosynthetic organs in utilizing light energy. The significant decrease in α after UV-B stress indicates that UV-B stress can reduce the efficiency of photosynthetic organs in utilizing light energy, while preapplication of ABA can reduce this effect. Ek is a parameter that responds to the plant’s ability to tolerate strong light, and the slight increase in Ek after UV-B stress may be the result of the plant initiating a photoprotective mechanism in order to minimize the damage caused by the stress.

To reflect the use of energy absorbed by photosystem II, we analyzed the curves of NPQ and qP with light intensity, as shown in Figure 2c. The NPQ curves reflected the ability of plants to dissipate excess light energy into heat, and it was found that the trends of the curves were consistent in different treatments, and the photoprotective ability of leaves increased with the increase in light intensity. In the unapplied ABA treatment group, the photoprotective ability of leaves did not change significantly after the application of UV-B stress. In the treatment group with pre-applied ABA, UV-B stress was able to reduce the photoprotective ability of leaves. The qP curve indicates the proportion of energy absorbed by photosystem II used to carry out photochemical reactions, which can reflect the level of photosynthetic activity. It can be observed from the curve that the photosynthetic activity of leaves decreased with increasing light intensity, and when PAR was 700 µmol (photon) m^−2^ s^−1^, the photosynthetic activity of the treatment group with UV-B stress applied was significantly lower than that of group A, which was irradiated by natural light only. Detection of chlorophyll fluorescence parameters using the Imaging-PAM chlorophyll fluorescence imaging system showed that Fv/Fm, α, and Ek clearly reflected the effects of different treatments on photosynthesis, UV-B stress reduced photosynthesis, and ABA alleviated the effects of UV-B stress on plant photosynthesis, which was consistent with the results of OJIP curves. Each photosynthetic parameter and its biological significance are shown in Table 1.

### 2.3. UV-B Stress Can Affect the Content of Photosynthesis-Related Proteins

**Figure 3:**
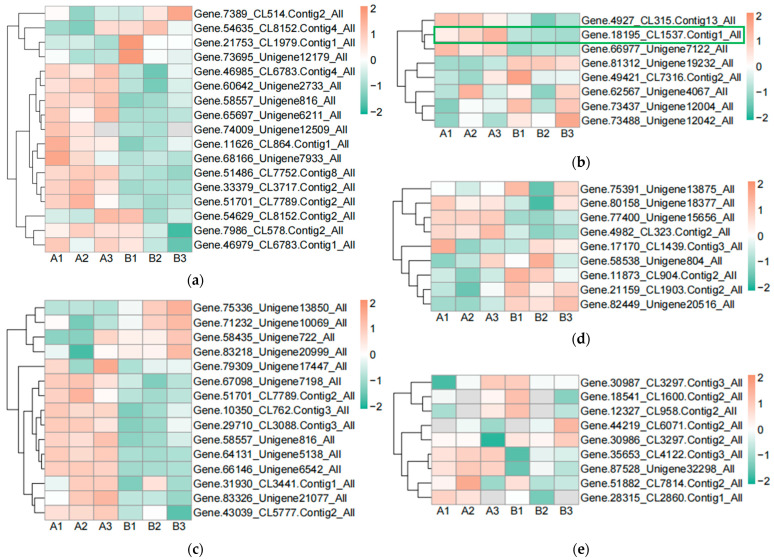

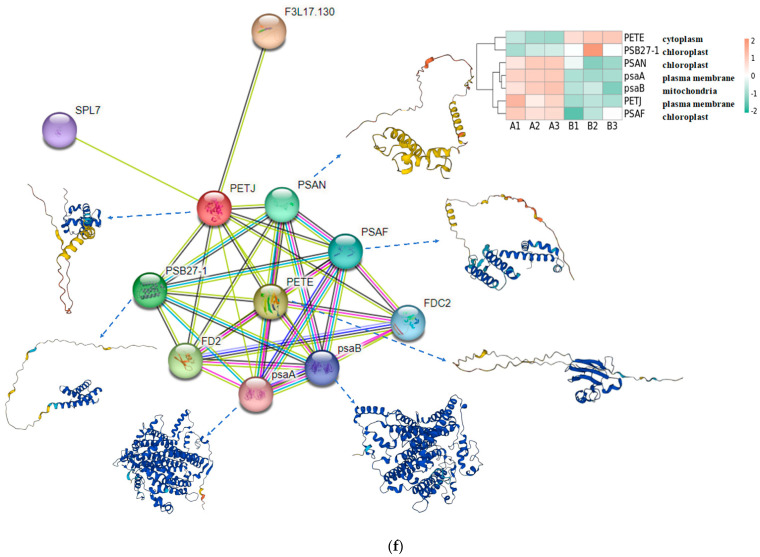
PETJ associated with photosynthesis was significantly altered in leaves of *R. chrysanthum* after UV-B stress. (**a**) Heatmap of protein expression associated with photosystem II; (**b**) Heatmap of protein expression associated with photosynthetic electron transport and cytochrome b6f complex; (**c**) Heatmap of protein expression associated with photosystem I; (**d**) Heatmap of protein expression associated with ATP synthase; (**e**) Heatmap of protein expression associated with antenna proteins (proteins were identified as complex-related based on their KEGG annotation information); (**f**) Protein interaction networks associated with PETJ (PETJ: cytochrome c6, photosynthesis proteins). A: control; B: UV-B treatment; PETE: Plastocyanin minor isoform, chloroplastic; PSB27-1: Photosystem II repair protein PSB27-H1, chloroplastic; PSAN: Photosystem I reaction center subunit N, chloroplastic; psaA: Photosystem I P700 chlorophyll a apoprotein A1; psaB: Photosystem I P700 chlorophyll a apoprotein A2; PSAF: Photosystem I reaction center subunit III, chloroplastic.

In order to find the effect of UV-B stress on proteins related to plant photosynthesis, we performed proteomics on leaves of *R. chrysanthum*. We screened proteins related to photosystem II, cytochrome b6f complex, photosystem I, ATP synthase, and antenna proteins, and presented their contents in the form of heat maps (Figure 3a–e, Table S2). Differential proteins were screened according to *p* < 0.05 and differential expression fold greater than 1.2 or differential expression fold less than 0.83. The content of Gene.18195_CL1537.Contig1_All in the photosynthetic electron transport was found to be significantly decreased after UV-B stress, which may be a key protein in response to UV-B stress during photosynthesis. The subcellular localization of Gene.18195_CL1537.Contig1_All protein showed that it is located in the plasma membrane, domain description showed that it has a cytochrome c-like domain, GO annotation showed that it has an electron carrier activity and can play a role in photosynthetic electron transport, and its gene name is PETJ. We looked for proteins interacting with PETJ in the STRING database, and as shown in Figure 3f, there are ten proteins capable of interacting with PETJ, and there are six related proteins in *R. chrysanthum*, of which PETE (PETE: Plastocyanin minor isoform) and PSB27-1 (PSB27-1: Photosystem II repair protein PSB27-H1) have elevated expression after UV-B stress, and psaA (psaA: Photosystem I P700 chlorophyll a apoprotein A1), PSAF (PSAF: Photosystem I reaction center subunit III), PSAN (PSAN: Photosystem I reaction center subunit N), and psaB (psaB: Photosystem I P700 chlorophyll a apoprotein A2) have reduced expression after UV-B stress. The expression, three-dimensional structure, and subcellular localization of the relevant proteins found in *R. chrysanthum* are presented in Figure 3f. PETJ, PETE, PSAF, psaA, and psaB are associated with electron transfer, and PSAN may play a role in mediating the binding of antenna complexes to photosystem I (PSI) reaction centers and core antennas. PSB27-1 may be involved in the repair of photodamaged PSII.

### 2.4. UV-B Stress Affects the Calvin Cycle in Leaves of R. chrysanthum

**Figure 4:**
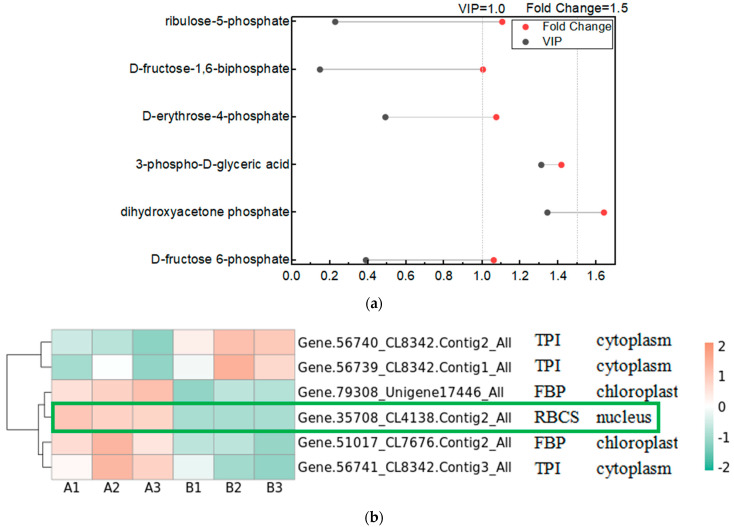

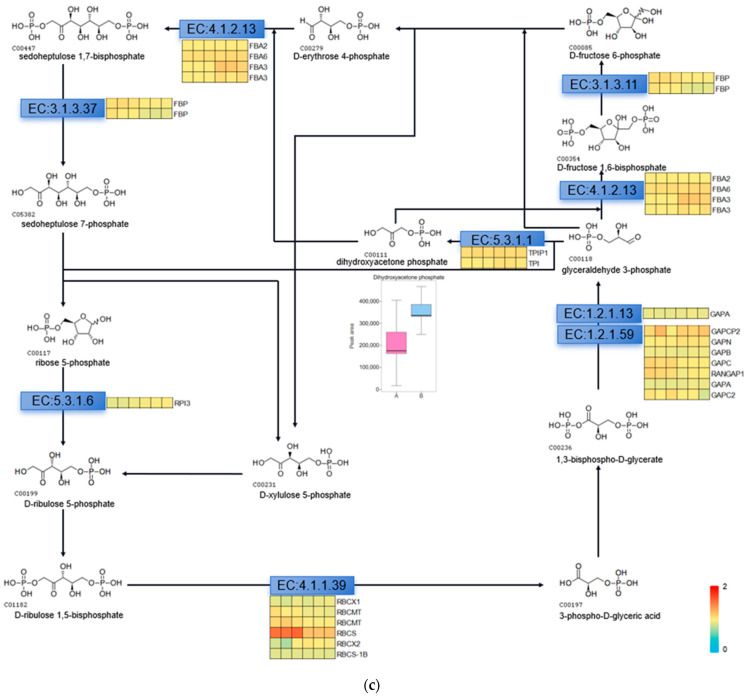
UV-B stress can affect the Calvin cycle in the leaves of *R. chrysanthum*. (**a**) Lollipop diagram of metabolites associated with the Calvin cycle identified by metabolomics; (**b**) Heat map of the expression of related enzymes in the Calvin cycle of *R. chrysanthum* under UV-B stress (TPI: triosephosphate isomerase; FBP: fructose-1,6-bisphosphatase; RBCS: ribulose-1,5-bisphosphate carboxylase); (**c**) Calvin cycle pathway under UV-B stress. A: control; B: UV-B treatment.

The Calvin cycle is an important reaction phase of plant photosynthesis, and we used metabolomics data to find the relevant metabolites in the pathway of the Calvin cycle, which are D-fructose 6-phosphate, dihydroxyacetone phosphate, 3-phospho-D-glyceric acid, D-erythrose-4-phosphate, D-fructose-1,6-biphosphate, and ribulose-5-phosphate. We used Variable Importance in Projection (VIP) greater than 1 and Fold Change (FC; experimental/control group) greater than 1.5 as the screening conditions, and screened the metabolite that changed significantly after UV-B stress as dihydroxyacetone phosphate, indicating that dihydroxyacetone phosphate is an important metabolite in the photosynthesis of *R. chrysanthum* in response to UV-B stress (Figure 4a). We used proteomics data to screen the enzymes regulated by light in the Calvin cycle of *R. chrysanthum*, and found that the enzymes related to the Calvin cycle in *R. chrysanthum* were triosephosphate isomerase (TPI), fructose-1,6-bisphosphatase (FBP), and ribulose-1,5-bisphosphate carboxylase (RBCS). Using *p* less than 0.05, and the fold of differential expression greater than 1.2 or less than 0.83 as the screening conditions, we found that the expression of RBCS was significantly reduced after UV-B stress, suggesting that UV-B stress can influence the expression of RBCS to regulate the Calvin cycle, thus reducing photosynthesis (Figure 4b). We show the Calvin cycle metabolic pathway and demonstrate the expression of relevant enzymes (Table S2) in the pathway in Figure 4c, and because dihydroxyacetone phosphate is a metabolite that changes significantly after UV-B stress, we have highlighted the expression of this substance in the figure with a box-and-line plot.

### 2.5. The G2-like Transcription Factor Family Is Able to Respond to UV-B Stress in R. chrysanthum Leaves

**Figure 5:**
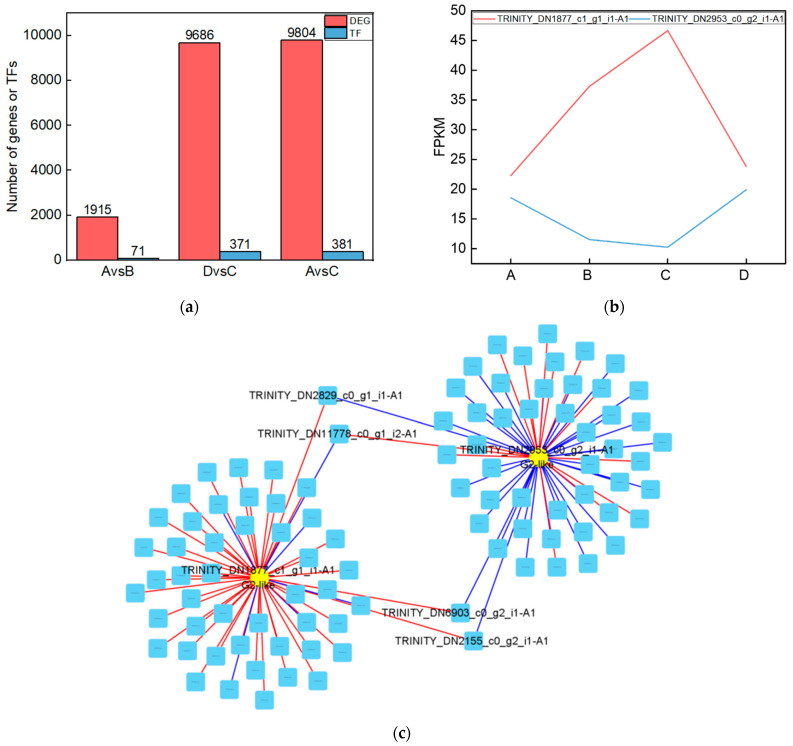

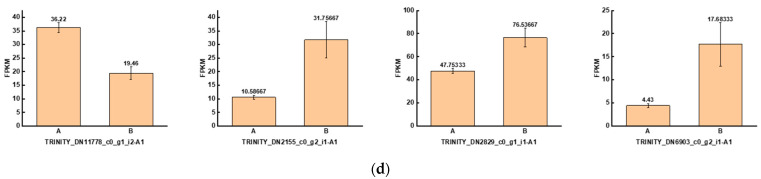
The G2-like transcription factor family is able to respond to UV-B stress in *R. chrysanthum* leaves. (**a**) Statistics on the number of differentially expressed genes and transcription factors among different treatment groups; (**b**) G2-like family member expression folding plot; (**c**) Correlation analysis of G2-like family members with other differentially expressed genes; (**d**) Bar graph of differentially expressed genes associated with all G2-like family members. Values are means ± S.E. (n = 3). FPKM: gene expression in each sample; A: control; B: UV-B treatment; C: UV-B + ABA treatment; D: ABA treatment.

To investigate the genes that were significantly altered after UV-B stress, we performed transcriptomic assays on leaves of the *R. chrysanthum*. At Qvalue < 0.05, genes with FC > 1 are up-regulated differentially expressed genes (DEGs) and FC < 1 are down-regulated DEGs. The number of DEGs among the comparison groups is shown in the red part of Figure 5a. Information about the DEGs in the Calvin cycle pathway in the AvsB group is shown in Table 2. We annotated the DEGs in each comparison group with transcription factors and counted the number of transcription factors as shown in the blue part of Figure 5a. As G2-like TFs play a role in regulating photosynthesis, this study screened the G2-like transcription factors in each comparison group, and the information is shown in Table 3. It was found that two members of G2-like were significantly altered after UV-B stress with or without the involvement of ABA. The line graphs of the expression of these two genes are shown in Figure 5b, and it was found that the expression patterns of these two genes were opposite. We predicted the binding sites for these two TFs as shown in Table S3. We correlated these two genes with the remaining 1913 DEGs after UV-B stress and screened the respective top fifty genes to draw a correlation network diagram (Figure 5c). The results revealed that TRINITY_DN2829_c0_g1_i1-A1, TRINITY_DN11778_c0_g1_i2-A1, TRINITY_DN6903_c0_g2_i1-A1, and TRINITY_DN2155_c0_g2_i1-A1 were highly correlated with these two G2-like TFs, and their expressions are shown in bar graphs (Figure 5d). These four genes may be the key genes in the response of *R. chrysanthum* leaves to UV-B stress in photosynthesis.

### 2.6. Intergroup Correlation Analysis

**Figure 6:**
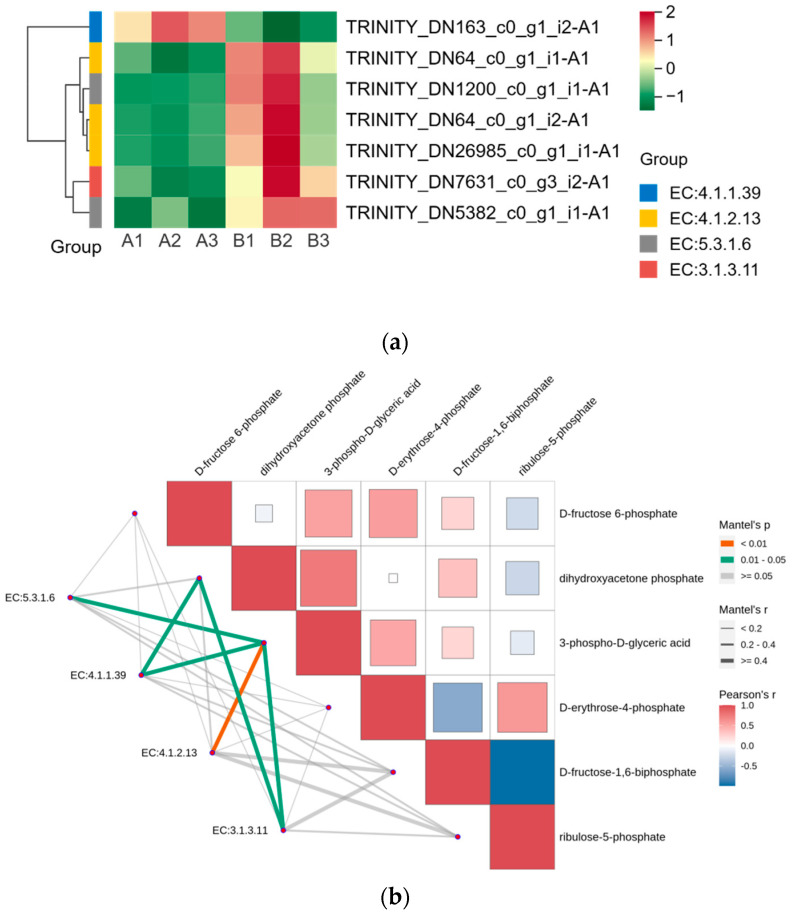
G2-like TFs strongly correlate with DEGs in the Calvin cycle under UV-B stress. (**a**) Heatmap of DEGs in the Calvin cycle; (**b**) Analysis of differentially expressed genes of the Calvin cycle pathway in correlation with metabolites: red and blue colors indicate the strength of the correlation, the line on the left side indicates the Mantel’s statistic, the thickness of the line reflects the correlation between differentially expressed genes (indicated by enzyme number) and metabolites, and the color of the line indicates the degree of significance. (EC5.3.1.6: ribose-5-phosphate isomerase; EC4.1.1.39: ribulose-bisphosphate carboxylase; EC4.1.2.13: fructose-1,6-bisphosphate aldolase; EC3.1.3.11: fructose-1,6-bisphosphatase).

We displayed the DEGs in the Calvin cycle as a heat map and found that these DEGs were able to encode four enzymes (Figure 6a). The gene encoding ribulose-1,5-bisphosphate carboxylase was expressed in the opposite pattern to the other six genes after UV-B stress. We correlated two members of the G2-like TF family with DEGs in the Calvin cycle, and the results are shown in Table 4, with correlation coefficients greater than 0.9, indicating that these two members of the G2-like TF family are strongly correlated with the Calvin cycle. Figure 4 reflects the upstream and downstream relationships between proteins and metabolites in the Calvin cycle pathway, and we correlated the DEGs (indicated by enzyme numbers) localized in this pathway with metabolites after UV-B stress, and the results are shown in Figure 6b. Metabolites significantly associated with ribulose-1,5-bisphosphate carboxylase and fructose-1,6-bisphosphatase are dihydroxyacetone phosphate and 3-phospho-D-glyceric acid. Fructose-1,6-bisphosphate aldolase and 3-phospho-D-glyceric acid are strongly correlated.

### 2.7. Comprehensive Multi-Omics Demonstrates the Photosynthetic Response to UV-B Stress in Leaves of R. chrysanthum

**Figure 7:**
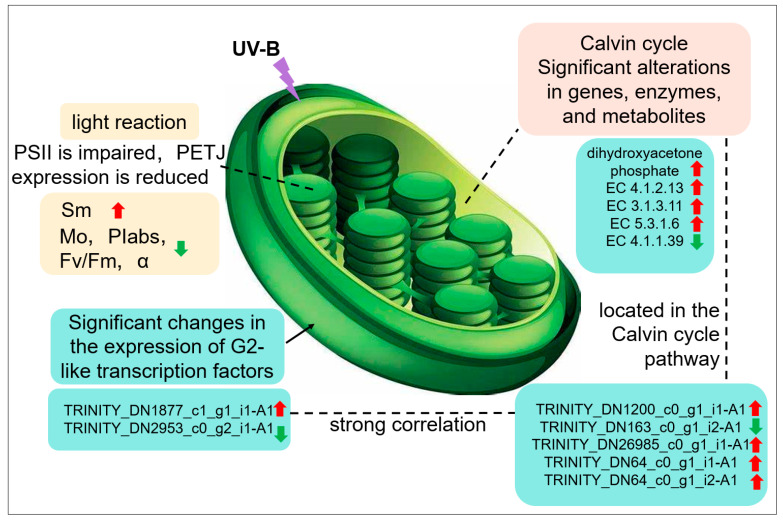
Response of photosynthesis to UV-B stress in leaves of *R. chrysanthum*.

It was found that UV-B stress was able to impair the photosynthetic system of *R. chrysanthum*. UV-B stress resulted in altered expression of photosystem-associated proteins, with a significant decrease in Gene.18195_CL1537.Contig1_All. The expression of enzymes associated with the Calvin cycle and genes encoding the enzymes was significantly altered, and the amount of dihydroxyacetone phosphate was significantly increased. Altered expression patterns of two members of the family of transcription factors annotated as G2-like; significant differences in the expression of genes related to photosynthesis. G2-like TFs can influence gene expression in chloroplasts affecting photosynthesis in *R. chrysanthum* in response to UV-B stress (Figure 7).

## 3. Discussion

Plants are exposed to an ever-changing environment, and although UV-B radiation (280–315 nm) represents a very small fraction of the solar radiation that reaches the Earth’s surface, it is also capable of causing significant effects on plants [20]. UV-B is capable of causing significant genetic damage to cellular DNA, breaking chemical bonds and producing highly reactive molecules that retard plant growth and development [21,22]. It has been shown that UV-B radiation is able to affect the content of substances in the leaves of *R. chrysanthum* and inhibit its photosynthesis at different histological levels [9]. However, there are fewer studies that combine physiological measurements of photosynthesis after stress on the *R. chrysanthum* with multi-omics. Therefore, in this study, we investigated the processes involved in photosynthesis in the *R. chrysanthum* using the Imaging-PAM chlorophyll fluorescence imaging system and the plant efficiency analyzer, and explored the possible roles of phytohormones; the changes of photosynthesis-related products under UV-B stress were further investigated using widely targeted metabolomics, proteomics and transcriptomics jointly, which provided information about *R. chrysanthum* for the study of plant responses under adverse environments.

Quantitative analysis of the fluorescence parameters at each point of the OJIP curve to understand the PSII primary photochemical reaction, electron transfer state, activity, and redox state of each reaction site of PSII enables assessment of the photosynthetic performance of plants. One study explored the effects of exogenous ascorbic acid on tomato seedlings under salt stress using OJIP rapid induction curves and analysis of fluorescence parameters to investigate the processes by which ascorbic acid affects the stability of the photosystem and the photosynthetic electron transport chain, as well as the relationship between energy use and consumption in the photosynthetic system [23]. It was shown that there was strong heterogeneity in the range of OJIP kinetic parameters and Q_A_ reoxidation parameters in plant leaves of different physiological states and treatments [24]. In the OJIP curves, normalization of the O-point revealed that the fluorescence value of group B with applied UV-B radiation was smaller than that of the other three groups, whereas the O-point was the fluorescence emitted by the primary quinone receptor Q_A_, the secondary quinone receptor Q_B_, and the plastoquinone (PQ) pool of the electron acceptors in PSII when they were in a fully oxidized state, suggesting that the UV-B radiation might reduce the number and activity of the pigments of the reaction centers. UV-B stress decreased PIabs in leaves of *R. chrysanthum*, reflecting damage to the photosynthetic apparatus. The decrease in Fm reflected the inhibition of water photolysis due to damage to the donor side of PSII as a result of UV-B stress, which led to a decrease in the electron transfer capacity of the acceptor side of PSII due to partial inhibition of the acceptor side of PSII before Q_A_ and a weakened ability to provide electrons downstream. The inhibition of Q_A_ reduction on the acceptor side of PSII led to a decrease in V_J_ (relative variable fluorescence intensity at point J). Although the PQ pool increased and the number of active reaction centers per unit area increased after UV-B stress, the damage to the donor side of PSII caused by UV-B stress resulted in the blockage of electron transfer and reduced photosynthesis. Although PIabs were reduced in group C *R. chrysanthum* compared with group A, they were slightly elevated compared with group B. ABA had a mitigating effect during the damage to the photosynthetic apparatus caused by UV-B stress. The changes in Fm, V_J_, and Sm all reflected that ABA could play a role in mitigating the damage caused by UV-B. It has been shown that high boron (B) stress reduces photosynthetic pigment content and photosynthetic rate in sugar beet, further reduces OEC activity on the electron donor side of PSII, and blocks electron transfer on the electron acceptor side, which is consistent with the results of this study [25].

The Imaging-PAM Maxi chlorophyll fluorescence detection system revealed that the Fv/Fm of the leaves of *R. chrysanthum* was significantly reduced after UV-B stress, whereas it was significantly reduced in group C compared with group A, and significantly increased in group C compared with group B. This indicates that UV-B stress can reduce the efficiency of the maximum light energy conversion of PSII and cause PSII to produce light damage, and ABA can alleviate the damage caused by UV-B to PSII. Decreased photochemical efficiency of PSII and PSI, elevated NPQ, and catabolism of core proteins related to photosynthesis in pea under drought stress indicated that drought stress resulted in a severe disruption of the photosynthetic machinery of the plant, and consistent results were obtained in this study [26]. The decrease in Fv/Fm after UV-B stress may be due to the loss of PSII activity caused by the degradation of D1 and D2 proteins, which is consistent with the damage to the donor side of PSII reflected by the OJIP curve. It was found that as PAR increased, NPQ also increased, excess light energy was dissipated into heat, and photoprotection of the photosynthetic system was enhanced. UV-B radiation was able to reduce NPQ regardless of the presence or absence of ABA, indicating that UV-B radiation is able to cause damage to the photosynthetic apparatus, whereas preapplication of ABA was able to increase the photoprotective capacity and reduce the damage of *R. chrysanthum* under UV-B stress. The decrease in qP with increasing PAR indicates that the proportion of open-state PSII reaction centers decreases, and photosynthetic activity decreases. Comparison of groups A and B showed that UV-B radiation could increase the proportion of open-state PSII reaction centers when PAR was low, and when PAR reached a certain value, the presence of UV-B radiation could reduce the proportion of open-state PSII reaction centers and decrease photosynthetic activity, and the results of group DvsC were consistent with those of group AvsB. Studies on *Hibiscus rosa-sinensis* showed that cold stress was able to cause inhibition of its PSII and a decrease in photosynthetic linear electron transport, which was manifested as a decrease in PSII photochemical efficiency and electron transport capacity [27]. Studies on cold-resistant cotton under cold stress showed that the photosynthesis system was severely damaged, inhibiting normal photosynthesis and causing photoinhibition [28]. Exogenous abscisic acid can alleviate the damage suffered by plants under abiotic stress, for example, studies on *Punica granatum* L. have shown that appropriate concentrations of abscisic acid can enhance some metabolic pathways (e.g., brassinosteroid synthesis, photosynthesis, etc.) and thus enhance the drought resistance of pomegranate [29].

The light reaction of photosynthesis occurs on the membrane of the vesicle-like body; light can excite the reaction center pigment to lose electrons, electron transfer to demagnesium chlorophyll, then electron transfer is transferred to PSI and, ultimately, transferred to the ferredoxin, and NADP^+^ to produce NADPH. Electron transfer is accompanied by the generation of a proton motive potential that drives ATP synthesis [30]. Proteomics data indicated that cyt c6 in photosynthetic electron transport was altered and PETJ content was significantly reduced after UV-B stress. Cytochrome c6 acts as an electron carrier between the membrane-bound cytochrome b6/f complex and PSI in oxygenic photosynthesis. Reduced expression of carrier proteins in the electron transport process under UV-B stress may be responsible for the limitation of photosynthesis in *R. chrysanthum* by UV-B radiation. A screening of light-regulated enzymes in the Calvin cycle revealed that the expression of ribulose-1,5-bisphosphate carboxylase was significantly reduced after UV-B stress, limiting photosynthesis in *R. chrysanthum*.

We determined the changes in the content of various metabolites in *R. chrysanthum* after UV-B stress using a widely targeted metabolomics assay, focusing on metabolites related to photosynthesis. We found that the content of dihydroxyacetone phosphate was significantly elevated in the Calvin cycle. Dihydroxyacetone phosphate is capable of reacting with glyceraldehyde 3-phosphate to form D-fructose 1,6-bisphosphate or with D-erythrose 4-phosphate to form sedoheptulose 1,7-bisphosphate (Figure 4). Previous studies have found that UV-B radiation enables *R. chrysanthum* to accumulate amino acids and carbohydrates [31]. Consistent results were obtained in this study.

We used transcriptomics to study the effects of UV-B stress on *R. chrysanthum*. It was found that some of the differentially expressed genes present among the different comparison groups could be identified as transcription factors. We screened the genes identified as G2-like transcription factors and found two members that were significantly changed across comparison groups. It has been shown that GLK genes regulate chloroplast development in different plant species [32]. Studies on *C. roseus* leaves have shown that silencing *CrGLK* reduces chlorophyll levels and decreases the expression of chloroplast-related genes [33]. The *GhGLK1* gene may be involved in regulating the response of cotton to drought and cold stress [34]. In addition, overexpression of *AhGLK1* was able to affect peanut morphogenesis and photosynthesis, thereby increasing its drought tolerance [35]. Four differential expression genes significantly related to two members of the G2-like family may affect the photosynthesis of *R. chrysanthum* by influencing the function of chloroplasts. G2-like TFs are strongly correlated with DEGs localized in the Calvin cycle pathway and may regulate the Calvin cycle process in *R. chrysanthum*. Analysis of the correlation between differentially expressed genes in the Calvin cycle pathway and metabolites after UV-B stress indicated that UV-B stress may contribute to changes in the expression of genes encoding enzymes, thereby regulating metabolite production.

Strong light can cause damage to plants, but dynamically changing antenna complexes to reduce the amount of incident light, dissipate excess light energy in the form of heat and fluorescence, and consume excess excitation energy to produce reactive oxygen byproducts is an effective measure to avoid light damage. When plants are subjected to stress, photosynthetic electron transport is first affected in response to stress; the metabolome, proteome, and transcriptome also change after stress, but the time at which the response occurs may be different, which explains why testing the same parameter at different times may produce opposite results. Multi-omics and physiological measurements have shown that G2-like transcription factors are able to regulate the Calvin cycle and thus photosynthesis under UV-B stress in *R. chrysanthum*.

## 4. Materials and Methods

### 4.1. Plant Materials and Treatments

*R. chrysanthum* collected from Changbai Mountain were cultured in an artificial climate chamber that simulated an alpine environment. The temperature of the artificial climate chamber was 16–18 °C (18 °C day/16 °C night), 50 µmol (photons) m^−2^ s^−1^ white fluorescent lamps, 14 h of light and 10 h of dark in a day-night cycle, and 60% relative humidity. *R. chrysanthum* group seedlings were carried in 1/4 MS medium.

Eight-month-old seedlings with uniform growth status were selected and divided into 4 groups, A, B, C, and D. Groups A and B were transferred into 1/4 MS medium, and groups C and D were transferred into 1/4 MS medium with ABA applied (100 µmol/L). Histocultured seedlings cultured for one week were used for the present study. Seedlings in groups A and B were subjected to physiological assays and multi-omics assays requiring fifteen biological replicates in each group, and seedlings in groups C and D were subjected to physiological assays and transcriptomic assays requiring nine biological replicates in each group, where each assay required three biological replicates for each group of samples.

**Figure 8:**
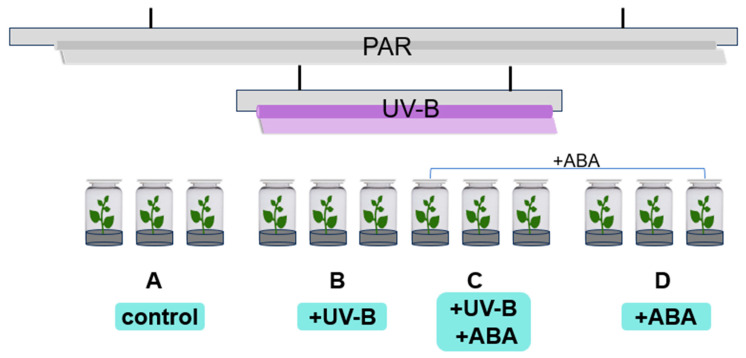
Schematic diagram of the radiation and grouping of *R. chrysanthum*.

The *R. chrysanthum* group seedlings were treated with radiation as shown in Figure 8. Groups A and D received PAR (wavelength 400–700 nm) irradiation, and Groups B and C received PAR + UV-B (280–315 nm) irradiation, and filters were placed on the culture flasks, which were wrapped in tinfoil and labeled. The fluorescent lamps and filters required for the experiment were the same as those of the previous experiment [8]. The effective irradiance of the samples was 50 µmol (photons) m^−2^ s^−1^ and 2.3 Wm^−2^ for PAR and UV-B, respectively, measured using the UV intensity meter (Sentry Optron-ICS Corp., ST-513, SHH, New Taipei City, China) and a light meter (TES Electrical Electronic Corp., Tes-1339 Light Meter Pro., Taipei, China). After UV-B radiation for 2 days (8 h/day), chlorophyll fluorescence was detected in the histocultured seedlings, and the leaves were collected and placed in liquid nitrogen for the subsequent multi-histology detection.

### 4.2. Detection of Chlorophyll Fluorescence

#### 4.2.1. Rapid Chlorophyll Fluorescence Induction Curve Determination

The fast chlorophyll fluorescence induction kinetic curve (OJIP curve) of leaves was determined by the Handy-PEA (Hansatech Instruments Ltd., King’s Lynn, UK) after fully dark-adapting the differently treated *R. chrysanthum* leaves for 30 min, the OJIP curve was induced by 3000 μmol·m^−2^ s^−1^ red light, and the determination time was 2 s. The OJIP curves were standardized for O-points, O-J segments, and O-P segments with reference to Strasser et al. [36]. Point O is the minimum fluorescence value, point K is the 300 μs fluorescence value, point J is the 2 ms fluorescence value, point I is the 30 ms fluorescence value, and point P is the maximum fluorescence value. The relevant parameters are calculated according to the following equation:

O-point standardization: F_t_/F_o_

O-P segment standardization: V_t_ = (F_t_ − F_o_)/(F_m_ − F_o_)

O-J segment standardization: W_t_ = (F_t_ − F_o_)/(F_J_ − F_o_)

ΔW_t_ = (W_t_)_treatment_ − (W_t_)_control_; ΔV_t_ = (V_t_)_treatment_ − (V_t_)_control_

#### 4.2.2. Slow-Phase Fluorescence Induction Curve Measurement

Chlorophyll fluorescence measurements were made using Imaging-PAM Maxi (Walz, Effeltrich version, Rohrdorf, Germany) after sufficient dark acclimatization (30 min) of the different treatments of *R. chrysanthum* group seedlings. The minimum fluorescence F_0_ was obtained by measuring light irradiation, then the saturation pulse was turned on, at which time the maximum fluorescence Fm was measured. The photochemical light was turned on to stimulate leaf photosynthesis, the saturation pulse light was turned on to obtain the maximum fluorescence under the light environment and, with the photochemical quenching and non-photochemical quenching under the light environment, the fluorescence yield was gradually reduced and reached the steady state, and the steady state fluorescence was measured.

### 4.3. Multi-Omics Assay of the Leaves of R. chrysanthum

#### 4.3.1. Widely Targeted Metabolomics Assays

The widely targeted metabolomics assay was performed by Wuhan Metware Biotechnology Co., Ltd. based on the UPLC-MS/MS detection platform and self-constructed database. The specific experimental procedure and reagents used were as described previously [37]. *R. chrysanthum* leaves were processed and stored in injection bottles for data collection. The data acquisition instrumentation system mainly consisted of Ultra Performance Liquid Chromatography (UPLC) (ExionLC™ AD, https://sciex.com.cn/) and Tandem mass spectrometry (MS/MS). Liquid phase conditions and mass spectrometry conditions are set according to company requirements. Based on the self-built database MWDB (metware database), substance characterization was performed based on secondary spectrum information, and metabolite quantification was performed using the multiple reaction monitoring (MRM) mode of triple quadrupole mass spectrometry.

#### 4.3.2. Transcriptomics Assays

The samples were melted on ice, centrifuged and mixed thoroughly, and appropriate amounts were taken for testing. The concentration of the samples was detected using an Agilent 2100 Bioanalyzer (Agilent, Santa Clara, CA, USA), the kit used was Agilent RNA6000 nano Reagents Part 1 (Agilent, Santa Clara, CA, USA), and the library type was DNBSEQStrand-SpecificTranscriptome. The quality of the tested samples met the criteria for library sequencing, and the success rate was high.

The mRNA with polyA tail was enriched with magnetic beads with OligodT; the obtained RNA was fragmented with interrupting buffer, and random N6 primers were used for reverse transcription, and then the cDNA duplex was synthesized to form double-stranded DNA. The synthesized double-stranded DNA was flattened and phosphorylated at the 5′ end, a sticky end with a protruding “A” was formed at the 3′ end, and a bulge-like junction with a protruding “T” at the 3′ end was joined. The ligated product was amplified by PCR with specific primers; the PCR product was heat denatured to a single strand, and then the single-stranded DNA was cyclized with a bridge primer to obtain a single-stranded circular DNA library. Up-sequencing was performed.

The raw data were filtered using SOAPnuke (v1.6.5), including reads containing connectors, reads with unknown base N content greater than 1%, and low quality reads [38]. The number of filtered reads was in the range of 42.34–43.46 M. The ratio of the number of bases with mass values greater than 20 to the total number of bases in the filtered reads was in the range of 97.83–98.43%, and the ratio of the number of bases with mass values greater than 30 to the total number of bases in the filtered reads was in the range of 92.4–94.51%. The de novo assembly of clean reads (removing PCR repeats to improve assembly efficiency) was performed using Trinity (v2.13.2), and then the assembled transcripts were clustered and de-redundant using CD-HIT (4.6) to obtain Unigene.

The ORFs of Unigene were detected using getorf (EMBOSS:6.5.7.0), then the ORFs were aligned to transcription factor protein structural domains using hmmsearch (data from TF), and then Unigene was competently characterized based on the transcription factor family profiles described by PlantTFDB. Clean reads were compared to reference gene sequences using Bowtie2 (v2.4.5), followed by RSEM to calculate gene and transcript expression levels [39,40].

#### 4.3.3. Proteomics Assays

The proteomics assay method was the same as that described by the previous authors [9]. The assay process was broadly divided into several processes: protein extraction, trypsin digestion, TMT labeling, HPLC classification, liquid chromatography-mass spectrometry tandem analysis, database search, and bioinformatics analysis.

Samples were removed from −80 °C, weighed into a liquid nitrogen pre-cooled mortar, and ground to powder with liquid nitrogen. Each group of samples was lysed by adding powder 4 times the volume of phenol extraction buffer (containing 10 mM dithiothreitol, 1% protease inhibitor, and 1% phosphatase inhibitor) and sonicated. An equal volume of Tris was added to equilibrate the phenol, centrifuged at 4 °C, 5500 g for 10 min, the supernatant was removed and precipitated overnight by adding 5 times the volume of 0.1 M ammonium acetate/methanol, and the protein precipitates were washed with methanol and acetone, respectively. The final precipitate was re-solubilized with 8 M urea and the protein concentration was determined using a BCA kit.

Dithiothreitol was added to the protein solution to give a final concentration of 5 mM and reduced at 56 °C for 30 min. Afterwards, iodoacetamide was added to make a final concentration of 11 mM and incubated for 15 min at room temperature away from light. Finally, the urea concentration of the samples was diluted to less than 2 M. Trypsin was added at a mass ratio of 1:50 (trypsin: protein) and digested at 37 °C overnight. Trypsin was then added at a mass ratio of 1:100 (trypsin: protein) and the enzymatic digestion was continued for 4 h.

Trypsinized peptides were desalted with Strata X C18 (Phenomenex, Torrance, CA, USA) and vacuum freeze-dried. Peptides were solubilized with 0.5 M TEAB and labeled according to the TMT kit operating instructions. Peptides were graded by high pH reverse HPLC on an Agilent 300Extend C18 column (Agilent, Santa Clara, CA, USA) (5 μm particle size, 4.6 mm inner diameter, 250 mm length).

Peptides were solubilized with liquid chromatography mobile phase A (0.1% (*v*/*v*) formic acid aqueous solution) and separated using an EASY-nLC 1000 ultra-high performance liquid chromatography system (Thermo Fisher Scientific, Waltham, MA, USA). The peptides were separated by an ultra-high performance liquid chromatography (UPLC) system and injected into an NSI ion source for ionization and then analyzed by Q Exactive^TM^ Plus (Thermo Fisher Scientific, Waltham, MA, USA). Secondary mass spectrometry data were retrieved using Maxquant (v1.5.2.8). The quantification method was set to TMT-6plex, and the FDR for protein identification and PSM identification were all set to 1%.

Gene Ontology (GO) annotations at the proteomic level were obtained from the UniProt-GOA database (http://www.ebi.ac.uk/GOA/, accessed on 10 March 2024). The submitted proteins were annotated for subcellular localization using the software wolfpsort (v.0.2) for predicting subcellular localization. Protein pathways were annotated using the KEGG (Kyoto Encyclopedia of Genes and Genomes) pathway database, and protein structural domains of identified proteins were annotated using InterProScan (v.5.14-53.0), a software based on protein sequence algorithms, and the corresponding InterPro structural domain database. Differential proteins were screened according to *p* < 0.05 and differential expression fold greater than 1.2 or differential expression fold less than 0.83.

### 4.4. Bioinformatics Analysis

On the basis of the Metware database established by Metware Biotechnology Co., Ltd. (Wuhan, China), the obtained data were further processed using the software Analyst 1.6.3. The differential metabolites (DMs) were filtered using the criteria VIP > 1, FC ≥ 1.5, and FC ≤ 0.67. At Q value < 0.05, genes with FC > 1 are up-regulated DEGs and FC < 1 are down-regulated DEGs. The screening criteria for differentially expressed proteins (DEPs) were FC ≥ 1.2 or FC ≤ 0.83 with a *p*-value < 0.05. Bar charts, radar charts, and lollipop charts were plotted using origin 2022, and heat maps were plotted using R package (v1.0.12), and the heat map tool was normalized using z-score. Protein network interaction maps and 3D structure maps were obtained from STRING (12.0) database, Calvin cycle pathway was constructed with reference to KEGG database (www.kegg.jp), and significance analysis was performed using SPSS (IBM SPSS Statistics 26). Transcription factor and differentially expressed gene correlation network was constructed by Cytoscape (3.10.1) platform, and transcription factor binding site prediction was performed using PlantTFDB (v5.0) database.

## 5. Conclusions

We combined physiological measurements and multi-omics to study photosynthesis in leaves of *R. chrysanthum* under UV-B stress. It was found that photosynthesis in *R. chrysanthum* was limited after UV-B stress, the donor side of PSII might be damaged, the expression of proteins related to the electron transport chain was reduced, and the expression of genes related to chloroplast photosynthesis was significantly altered. From the results of physiological assays, it was found that preapplication of ABA may alleviate the effects of UV-B stress on photosynthesis in *R. chrysanthum* and reduce the damage to the photosystem. The results showed that the light response was weakened under UV-B stress in *R. chrysanthum*, and the G2-like TFs were significantly altered, which regulated the expression of the relevant genes in the Calvin cycle pathway and reduced photosynthesis. This paper provides insights into the study of photosynthesis in plants under stressful environments and provides an understanding of subsequent measures to enhance photosynthesis in plants under stressful environments to improve yield.

## Figures and Tables

**Table 1:** Parameters related to photosynthesis.

Parameter	Biological Significance
Wk	Relative variable fluorescence intensity at point K
ψ_0_	Probability of captured energy transferring electrons after Q_A_
M_0_	Maximum rate at which Q_A_ is reduced
RC/CS_0_	Number of reaction centers per unit area (at t = 0)
Ψ_E0_	Probability of absorbed light energy transferring electrons beyond Q_A_
TR_0_/CS_0_	Light energy captured per unit area (at t = 0)
ET_0_/CS_0_	Quantum yield of electron transfer per unit area (at t = 0)
TR_0_/RC	Energy captured by unit reaction center for reduction of Q_A_ (at t = 0)
ET_0_/RC	Energy captured for electron transfer per unit reaction center (at t = 0)
Sm	Area under the OJIP curve
PIabs	Composite performance index (absorption-based)
F_0_	Minimum fluorescence intensity
Fm	Maximum fluorescence intensity
Y(NO)	The part of the excitation energy absorbed by photosystem II that is passively dissipated as heat and emits fluorescence is mainly contributed by the photosystem II reaction center in the closed state
ΦPSII	Actual light energy conversion efficiency of photosystem II
ETRmax	Potential maximum relative electron transfer rate
α	Initial slope of the fast light curve
Ek	Ability to withstand bright light
NPQ	Non-photochemical quenching
qP	Photochemical quenching
Fv/Fm	Maximum photosynthetic efficiency of photosystem II

**Table 2:** Information on differentially expressed genes in the Calvin cycle pathway.

Gene ID	Qvalue (AvsB)	log2 (B/A)	Pvalue (AvsB)	Kegg Orthology
TRINITY_DN26985_c0_g1_i1-A1	0.002654807	1.513842229	0.0000299	EC:4.1.2.13
TRINITY_DN64_c0_g1_i1-A1	0.026921138	0.738460022	0.0007902	EC:4.1.2.13
TRINITY_DN64_c0_g1_i2-A1	0.005398428	1.417834419	0.0000761	EC:4.1.2.13
TRINITY_DN1200_c0_g1_i1-A1	0.038090126	0.992662495	0.0013437	EC:5.3.1.6
TRINITY_DN5382_c0_g1_i1-A1	0.029219008	0.728632982	0.0008935	EC:5.3.1.6
TRINITY_DN163_c0_g1_i2-A1	0.033252883	−0.716435144	0.0010878	EC:4.1.1.39
TRINITY_DN7631_c0_g3_i2-A1	0.005903226	1.076873143	0.0000853	EC:3.1.3.11

**Table 3:** Information on G2-like transcription factor family members that changed significantly in each comparison group.

Gene ID	log2 (B/A)	Qvalue (AvsB)	log2 (C/D)	Qvalue (DvsC)	log2 (C/A)	Qvalue (AvsC)
TRINITY_DN1877_c1_g1_i1-A1	0.86039	0.01941	1.19700	0.00000	1.29359	0.00000
TRINITY_DN2953_c0_g2_i1-A1	−0.60210	0.03757	−0.73871	0.00001	−0.64054	0.00066
TRINITY_DN19661_c0_g1_i1-A1	-	-	−1.75354	0.00001	-	-
TRINITY_DN2735_c0_g1_i1-A1	-	-	−0.72304	0.02554	-	-
TRINITY_DN8140_c0_g2_i1-A1	-	-	−1.04692	0.00724	-	-
TRINITY_DN8663_c0_g1_i1-A1	-	-	−0.87317	0.00001	-	-
TRINITY_DN1054_c1_g2_i2-A1	-	-	-	-	−0.56104	0.00050
TRINITY_DN19661_c0_g1_i1-A1	-	-	-	-	−1.13608	0.00476
TRINITY_DN2735_c0_g1_i1-A1	-	-	-	-	−0.87518	0.00190
TRINITY_DN8140_c0_g2_i1-A1	-	-	-	-	−0.79315	0.04115

**Table 4:** Statistics on the correlation of G2-like TF family members with DEGs in the Calvin cycle pathway.

G2-like TF Family Members	DEGs in the Calvin Cycle Pathway	PCC	*p*-Value
TRINITY_DN1877_c1_g1_i1-A1	TRINITY_DN1200_c0_g1_i1-A1	0.933296371	0.006525666
	TRINITY_DN163_c0_g1_i2-A1	−0.963016176	0.002026412
	TRINITY_DN26985_c0_g1_i1-A1	0.976969538	0.000789496
	TRINITY_DN64_c0_g1_i1-A1	0.988595464	0.000194354
	TRINITY_DN64_c0_g1_i2-A1	0.964488272	0.001869233
TRINITY_DN2953_c0_g2_i1-A1	TRINITY_DN1200_c0_g1_i1-A1	−0.951440039	0.003479851
	TRINITY_DN163_c0_g1_i2-A1	0.958290919	0.002573192
	TRINITY_DN26985_c0_g1_i1-A1	−0.982354731	0.000464286
	TRINITY_DN64_c0_g1_i1-A1	−0.968181729	0.001502497
	TRINITY_DN64_c0_g1_i2-A1	−0.971121463	0.001238913

## Data Availability

The data used in this study are available from the corresponding author on submission of a reasonable request.

## Supplementary Materials

The following supporting information can be downloaded at: https://www.mdpi.com/article/10.3390/plants13131856/s1, Figure S1: Normalization of OJIP curves in leaves of *R. chrysanthum* after UV-B stress; Table S1: Raw data for representative fluorescence parameters; Table S2: Proteomic assay information; Table S3: TRINITY_DN1877_c1_g1_i1-A1 and TRINITY_DN2953_c0_g2_i1-A1 binding site prediction.

## References

[1] Liaqat W., Altaf M.T., Barutçular C., Nawaz H., Ullah I., Basit A., Mohamed H.I. (2023). Ultraviolet-B radiation in relation to agriculture in the context of climate change: A review. Cereal Res. Commun..

[2] Barnes P.W., Robson T.M., Neale P.J., Williamson C.E., Zepp R.G., Madronich S., Wilson S.R., Andrady A.L., Heikkilä A.M., Bernhard G.H. (2022). Environmental effects of stratospheric ozone depletion, UV radiation, and interactions with climate change: UNEP environmental effects assessment panel, update 2021. Photochem. Photobiol. Sci..

[3] Robson T.M., Klem K., Urban O., Jansen M.A. (2015). Re-interpreting plant morphological responses to UV-B radiation. Plant Cell Environ..

[4] Chen Z., Dong Y., Huang X. (2022). Plant responses to UV-B radiation: Signaling, acclimation and stress tolerance. Stress Biol..

[5] Kataria S., Jajoo A., Guruprasad K.N. (2014). Impact of increasing Ultraviolet-B (UV-B) radiation on photosynthetic processes. J. Photochem. Photobiology. B Biol..

[6] Liu W., Giuriani G., Havlikova A., Li D., Lamont D.J., Neugart S., Velanis C.N., Petersen J., Hoecker U., Christie J.M. (2024). Phosphorylation of *Arabidopsis* UVR8 photoreceptor modulates protein interactions and responses to UV-B radiation. Nat. Commun..

[7] Zhou X., Chen S., Wu H., Yang Y., Xu H. (2017). Biochemical and proteomics analyses of antioxidant enzymes reveal the potential stress tolerance in *Rhododendron chrysanthum* Pall. Biol. Direct.

[8] Gong F., Yu W., Zeng Q., Dong J., Cao K., Xu H., Zhou X. (2023). *Rhododendron chrysanthum*’s primary metabolites are converted to phenolics more quickly when exposed to UV-B radiation. Biomolecules.

[9] Liu M., Sun Q., Cao K., Xu H., Zhou X. (2023). Acetylated proteomics of UV-B stress-responsive in Photosystem II of *Rhododendron chrysanthum*. Cells.

[10] Kirchhoff H. (2019). Chloroplast ultrastructure in plants. New Phytol..

[11] Liu F., Xu Y., Han G., Zhou L., Ali A., Zhu S., Li X. (2016). Molecular evolution and genetic variation of G2-like transcription factor genes in maize. PLoS ONE.

[12] Waters M.T., Wang P., Korkaric M., Capper R.G., Saunders N.J., Langdale J.A. (2009). GLK transcription factors coordinate expression of the photosynthetic apparatus in *Arabidopsis*. Plant Cell.

[13] Waters M.T., Langdale J.A. (2009). The making of a chloroplast. EMBO J..

[14] Baslam M., Mitsui T., Hodges M., Priesack E., Herritt M.T., Aranjuelo I., Sanz-Sáez Á. (2020). Photosynthesis in a changing global climate: Scaling up and scaling down in crops. Front. Plant Sci..

[15] Wu J., Hu J., Wang L., Zhao L., Ma F. (2021). Responses of *Phragmites australis* to copper stress: A combined analysis of plant morphology, physiology and proteomics. Plant Biol..

[16] Baker C.R., Patel-Tupper D., Cole B.J., Ching L.G., Dautermann O., Kelikian A.C., Allison C., Pedraza J., Sievert J., Bilbao A. (2023). Metabolomic, photoprotective, and photosynthetic acclimatory responses to post-flowering drought in sorghum. Plant Direct.

[17] Strauss A.J., Krüger G.H.J., Strasser R.J., Heerden P.D.R.V. (2006). Ranking of dark chilling tolerance in soybean genotypes probed by the chlorophyll a fluorescence transient O-J-I-P. Environ. Exp. Bot..

[18] Ren H., Lu Y., Tang Y., Ren P., Tang H., Chen Q., Kuang P., Huang R., Zhu W., Chen K. (2024). Photosynthetic responses of *Racomitrium japonicum* L. to Strontium Stress evaluated through chlorophyll a fluorescence OJIP transient analysis. Plants.

[19] Shapiguzov A., Kangasjärvi J. (2022). Studying plant stress reactions in vivo by PAM Chlorophyll Fluorescence Imaging. Methods Mol. Biol..

[20] Dotto M., Casati P. (2017). Developmental reprogramming by UV-B radiation in plants. Plant Sci..

[21] Gill S.S., Anjum N.A., Gill R., Jha M., Tuteja N. (2015). DNA damage and repair in plants under ultraviolet and ionizing radiations. Sci. World J..

[22] Tossi V.E., Regalado J.J., Iannicelli J., Laino L.E., Burrieza H.P., Escandón A.S., Pitta-Álvarez S.I. (2019). Beyond *Arabidopsis*: Differential UV-B response mediated by *UVR8* in diverse species. Front. Plant Sci..

[23] Chen X., Zhou Y., Cong Y., Zhu P., Xing J., Cui J., Xu W., Shi Q., Diao M., Liu H.Y. (2021). Ascorbic acid-induced photosynthetic adaptability of processing tomatoes to salt stress probed by fast OJIP fluorescence rise. Front. Plant Sci..

[24] Küpper H., Benedikty Z., Morina F., Andresen E., Mishra A., Trtílek M. (2019). Analysis of OJIP chlorophyll fluorescence kinetics and Q(A) reoxidation kinetics by direct fast imaging. Plant Physiol..

[25] Huo J., Song B., Riaz M., Song X., Li J., Liu H., Huang W., Jia Q., Wu W. (2022). High boron stress leads to sugar beet (*Beta vulgaris* L.) toxicity by disrupting photosystem II. Ecotoxicol. Environ. Saf..

[26] Pandey J., Devadasu E., Saini D., Dhokne K., Marriboina S., Raghavendra A.S., Subramanyam R. (2023). Reversible changes in structure and function of photosynthetic apparatus of pea (*Pisum sativum*) leaves under drought stress. Plant J. Cell Mol. Biol..

[27] Paredes M., Quiles M.J. (2015). The effects of cold stress on photosynthesis in *Hibiscus* plants. PLoS ONE.

[28] Li Y., Zhu J., Xu J., Zhang X., Xie Z., Li Z. (2024). Effect of cold stress on photosynthetic physiological characteristics and molecular mechanism analysis in cold-resistant cotton (ZM36) seedlings. Front. Plant Sci..

[29] Qian J., Zhang X., Yan Y., Wang N., Ge W., Zhou Q., Yang Y. (2020). Unravelling the molecular mechanisms of abscisic acid-mediated drought-stress alleviation in pomegranate (*Punica granatum* L.). Plant Physiol. Biochem. PPB.

[30] Rochaix J.D. (2011). Regulation of photosynthetic electron transport. Biochim. Et Biophys. Acta.

[31] Sun Q., Liu M., Cao K., Xu H., Zhou X. (2022). UV-B irradiation to amino acids and carbohydrate metabolism in *Rhododendron chrysanthum* leaves by coupling deep transcriptome and metabolome analysis. Plants.

[32] Fitter D.W., Martin D.J., Copley M.J., Scotland R.W., Langdale J.A. (2002). GLK gene pairs regulate chloroplast development in diverse plant species. Plant J. Cell Mol. Biol..

[33] Cole-Osborn L.F., McCallan S.A., Prifti O., Abu R., Sjoelund V., Lee-Parsons C.W.T. (2024). The role of the Golden2-like (GLK) transcription factor in regulating terpenoid indole alkaloid biosynthesis in *Catharanthus roseus*. Plant Cell Rep..

[34] Liu J., Mehari T.G., Xu Y., Umer M.J., Hou Y., Wang Y., Peng R., Wang K., Cai X., Zhou Z. (2021). *GhGLK1* a key candidate gene from GARP family enhances cold and drought stress tolerance in cotton. Front. Plant Sci..

[35] Liu X., Li L., Li M., Su L., Lian S., Zhang B., Li X., Ge K., Li L. (2018). *AhGLK1* affects chlorophyll biosynthesis and photosynthesis in peanut leaves during recovery from drought. Sci. Rep..

[36] Strasser R.J., Tsimilli-Michael M., Qiang S., Goltsev V. (2010). Simultaneous in vivo recording of prompt and delayed fluorescence and 820-nm reflection changes during drying and after rehydration of the resurrection plant *Haberlea rhodopensis*. Biochim. Et Biophys. Acta.

[37] Zhou X., Gong F., Dong J., Lin X., Cao K., Xu H., Zhou X. (2024). Abscisic acid affects phenolic acid content to increase tolerance to UV-B stress in *Rhododendron chrysanthum* Pall. Int. J. Mol. Sci..

[38] Chen Y., Chen Y., Shi C., Huang Z., Zhang Y., Li S., Li Y., Ye J., Yu C., Li Z. (2018). SOAPnuke: A MapReduce acceleration-supported software for integrated quality control and preprocessing of high-throughput sequencing data. GigaScience.

[39] Langmead B., Salzberg S.L. (2012). Fast gapped-read alignment with Bowtie 2. Nat. Methods.

[40] Li B., Dewey C.N. (2011). RSEM: Accurate transcript quantification from RNA-Seq data with or without a reference genome. BMC Bioinform..

