# Supplementary material for: Golden 2-like Transcription Factors Regulate Photosynthesis under UV-B Stress by Regulating the Calvin Cycle
Source: Plants (Basel). 2024 Jul 5;13(13):1856. doi: 10.3390/plants13131856 (PMC11243960; doi:10.3390/plants13131856)
Supplement: Supplementary file 1 [file plants-13-01856-s001.zip › Figure S1+Table S1+Table S3.pdf]

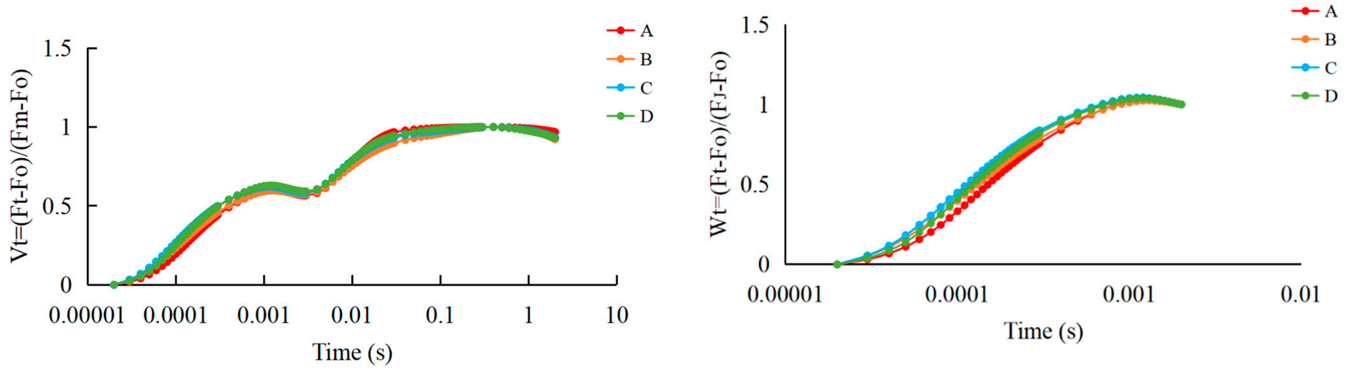

(**a**) (**b**)

Figure S1. Normalization of OJIP curves in leaves of *R. chrysanthum* after UV-B stress. (a) Normalized Vt curve at point O-P; (b) Normalized Wt curve at point O-J. A: control; B: UV-B treatment; C: UV-B+ABA treatment; D: ABA treatment.

Table S1. Raw data for representative fluorescence parameters.

	**A**	**B**	**C**	**D**
Wk	0.758688525	0.788018433	0.838028169	0.821247892
$_{\varphi}0$	0.4006	0.4139	0.4024	0.3805
MO	1.856670342	1.853137517	2.005850397	2.058386937
RC/CS0	154.7842299	196.8817775	178.2864326	132.5210824
$_{\varphi}E0$	0.33001428	0.2992497	0.30175976	0.29758905
Fv/Fo	4.675257732	2.609756098	2.998746867	3.589698046
TRo/CSo	479.4496	622.4797	598.4375	440.334
ETo/CSo	192.0618	257.6351	240.8254	167.5491
TRo/RC	2.5812	2.6348	2.7972	2.7689
ETo/RC	1.034	1.0905	1.1257	1.0536
Fv/Fm	0.824	0.723	0.75	0.782
Sm	6.7217	14.524	10.5642	8.1296
PI abs	0.997	0.506	0.541	0.623

Table S3. TRINITY_DN1877_c1_g1_i1-A1 and TRINITY_DN2953_c0_g2_i1-A1 binding site prediction.

sequence name	pattern name	Family	start	stop	strand	score	p-value	q-value	matched sequence
TRINITY_DN1877_c1_g1_i1-A1	AT1G18960	MYB_related	509	530	+	17.6301	0.000000165	0.000421	GAAGCCCATCACCAACAGTGCC
	AT1G18960	MYB_related	820	841	+	17.6301	0.000000165	0.000421	GAAGCCCATCACCAACAGTGCC
	AT1G22640	MYB	1593	1603	+	16.9255	0.000000181	0.00101	GGGTAGGTAGA
	AT5G18090	B3	479	493	-	15.6438	0.000000328	0.000893	GATGATGAAGAGGGA
	AT5G18090	B3	790	804	-	15.6438	0.000000328	0.000893	GATGATGAAGAGGGA
	AT5G49330	MYB	1593	1602	+	16.3269	0.000000545	0.003	GGGTAGGTAG
	AT5G12870	MYB	1593	1602	+	15.3365	0.000000679	0.00376	GGGTAGGTAG
	AT1G03800	ERF	1072	1092	-	16.8594	0.000000801	0.00189	GGTGGCACTAGCTCCGCCACT
	AT1G03800	ERF	1383	1403	-	16.8594	0.000000801	0.00189	GGTGGCACTAGCTCCGCCACT
	AT3G57600	ERF	1088	1098	+	16.9688	0.000000979	0.00228	CCACCACCGCA
TRINITY_DN2953_c0_g2_i1-A1	AT2G01930	BBR-BPC	4	27	-	23.5781	0.0000000028	1.50E-05	GAGAGGGGGAGAGAGAAGGAATGA

	AT2G01930	BBR-BPC	2	25	-	19.3594	0.0000000241	6.37E-05	GAGGGGGAGAGAGAAGGAATGACA
	AT5G42520	BBR-BPC	7	27	+	18.9589	0.0000000346	0.000185	TTCCTTCTCTCTCCCCCTCTC
	AT2G01930	BBR-BPC	1529	1552	+	18.4688	0.0000000370	6.52E-05	GAGAGGAAAAGAAAAAGAAAGAGT
	AT2G01930	BBR-BPC	12	35	-	17.7188	0.0000000527	6.96E-05	TTGAGGTTGAGAGGGGGAGAGAGA
	AT5G17430	AP2	1531	1550	+	19.1519	0.0000000643	0.000347	GAGGAAAAGAAAAAGAAAGA
	AT2G01930	BBR-BPC	1527	1550	+	16.3125	0.0000001000	0.000106	CAGAGAGGAAAAGAAAAAGAAAGA
	AT5G42520	BBR-BPC	13	33	+	15.9589	0.0000001310	0.00035	CTCTCTCCCCCTCTCAACCTC
	AT4G38000	Dof	1533	1560	-	17.9688	0.0000001560	0.000846	TCCTTTTTACTCTTTCTTTTTCTTTTCC
	AT4G38910	BBR-BPC	1534	1563	+	-16.6203	0.0000001790	0.000509	GAAAAGAAAAAGAAAGAGTAAAAAGGAGAG
